# Prediction and prevention of preeclampsia: Number 1 – January 2023

**DOI:** 10.1055/s-0043-1763495

**Published:** 2023-03-06

**Authors:** Fernando Maia Peixoto-Filho, Fabricio da Silva Costa, Sergio Kobayashi, Patricia El Beitune, Adriana Gualda Garrido, Anselmo Verlangieri Carmo, Guilherme de Castro Rezende, Heron Werner Junior, Joffre Amin Junior, Jorge Roberto Di Tommaso Leão, Luciano Marcondes Machado Nardozza, Luiz Eduardo Machado, Manoel Alfredo Curvelo Sarno, Pedro Pires Ferreira Neto, Eduardo Becker Júnior

**Affiliations:** 1 Universidade Federal Fluminese, Niteroi, RJ, Brazil; 2 Maternal Fetal Medicine Unit, Gold Coast University Hospital, Southport, Queensland, Australia; 3 Universidade Federal de São Paulo, São Paulo, SP, Brazil; 4 Universidade Federal de Ciências da Saúde de Porto Alegre, Porto Alegre, RS, Brazil; 5 Universidade de Brasília, Brasília, DF, Brazil; 6 Universidade Federal de Mato Grosso, Cuiabá, MT, Brazil; 7 Universidade Federal de Minas Gerais, Belo Horizonte, MG, Brazil; 8 Universidade Federal do Rio de Janeiro, Rio de Janeiro, RJ, Brazil; 9 Universidade Federal do Rio de Janeiro, Rio de Janeiro, RJ, Brazil; 10 Universidade do Estado do Amazonas, Manaus, AM, Brazil; 11 Universidade Federal de São Paulo, São Paulo, SP, Brazil; 12 Escola Bahiana de Medicina e Saúde Pública, Salvador, BA, Brazil; 13 Universidade Federal da Bahia, Salvador, BA, Brazil; 14 Universidade de Pernambuco, Recife, PE, Brazil; 15 Pontifícia Universidade Católica do Rio Grande do Sul, Porto Alegre, RS, Brazil

## Keypoints

- Preeclampsia (PE) is an important cause of maternal and perinatal mortality worldwide, accounts for 10% to 15% of direct maternal deaths, and 99% of these deaths are in low-income countries.
- Preeclampsia is defined as systolic blood pressure of ≥140 mmHg and/or diastolic blood pressure of ≥90 mmHg on at least two occasions, measured four hours apart in previously normotensive women, and is accompanied by one or more of the following new-onset conditions after 20 weeks' gestation: (1) proteinuria, (2) evidence of other maternal organ dysfunction, or (3) uteroplacental dysfunction.
- Preeclampsia is classified into: (1) early PE (delivery < 34+0 weeks' gestation); (2) preterm PE (delivery < 37+0 weeks' gestation); (3) late-onset PE (delivery ≥ 34+0 weeks' gestation); (4) term PE (delivery ≥ 37+0 weeks' gestation).
- In Brazil, the incidence of PE varies from 1.5% to 7%; of preterm PE is 2% and of eclampsia is 0.6%. However, these statistics are likely to be underestimated and vary according to the region studied.
- Screening strategies for PE vary depending on the parameters used, pre-test risk, outcome stratification, and the gestational age at which screening is performed. However, there is consensus in the literature that no single-parameter screening test has been shown to adjust the preexisting maternal risk for PE with sufficient specificity and sensitivity for clinical use.

## Recommendations

- Screening of all pregnant women is recommended to identify those at higher risk for PE so that they can receive preventive measures and greater maternal-fetal surveillance during pregnancy.
- The best strategies for screening PE involve several parameters in combination from a risk calculation algorithm. The decision on which maternal and fetal parameters should be included depends on the availability of resources in different settings.
- The best risk calculation strategy for PE uses a combination of maternal factors, mean arterial pressure, mean uterine artery pulsatility index, maternal serum pregnancy-associated plasma protein A (PAPP-A) or placental growth factor (PlGF) at 11-14 weeks' gestation using the concurrent risk model developed by the Fetal Medicine Foundation.
- At a risk cutoff of 1 in 100 for PE, the positive screening rate was 10%, and the detection rates of preterm and full-term PE were approximately 69% and 40%, respectively. Thus, these patients should be classified as high risk for PE.
- Patients at high risk for PE, i.e. risk ≥ 1:100 at 11-14 weeks' gestation, should start using acetylsalicylic acid (ASA) at a dose ≥ 100 mg, ideally 150 mg. Use should be started before 16 weeks and continued until 36 weeks.

## Background

Preeclampsia (PE) is an important cause of maternal and perinatal mortality worldwide. It represents 10-15% of direct maternal deaths and 99% of these deaths occur in low-income countries. 1 A systematic review by Abalos et al. in 2013, 2 showed an incidence ranging from 1.2% to 4.2% for PE and 0.1% to 2.7% for eclampsia. The highest rates were identified in regions of lower socioeconomic development. In Brazil, the incidence of PE ranges from 1.5% to 7%, 2 3 that of preterm PE is 2% 3 and of eclampsia is 0.6%. 2 However, these statistics may be underestimated and vary according to the region studied.

Although the pathogenesis of PE remains unknown, the most accepted theory suggests a two-stage process. In the first stage, there would be a superficial invasion of the trophoblast, resulting in inadequate remodeling of the spiral arteries, which would lead to the second stage that involves the maternal response to endothelial dysfunction and an imbalance between angiogenic and antiangiogenic factors, resulting in the clinical features of this condition. 4 5 6 Although the placenta plays an essential role in the development of PE, evidence suggests that the maternal cardiovascular system contributes significantly to the disorder. 7

According to the International Society for the Study of Hypertension in Pregnancy (ISSHP), PE is defined as systolic blood pressure of ≥140 mmHg and/or diastolic blood pressure of ≥90 mmHg on at least two occasions, measured at four-hour intervals in previously normotensive women, and is accompanied by one or more of the following new-onset conditions after 20 weeks' gestation: (1) proteinuria, (2) evidence of other maternal organ dysfunction, or (3) uteroplacental dysfunction. With regard to classification, PE can still be subclassified into: (1) early PE (delivery < 34+0 weeks' gestation); (2) preterm PE (delivery < 37+0 weeks' gestation); (3) late-onset PE (delivery ≥ 34+0 weeks' gestation); (4) full-term PE (delivery ≥ 37+0 weeks' gestation). 8 The capacity of screening tests, the management and maternal and perinatal mortality will vary according to this classification. It is important to identify women at higher risk for PE so they can receive preventive measures and greater maternal and fetal surveillance during pregnancy. 6

## What are the parameters and strategies for predicting preeclampsia?

The screening strategies for PE described in the literature vary according to the parameters used, the pre-test risk, the stratification of the result and the gestational age at which the screening is performed. However, there is consensus in the literature that no single-parameter screening test has shown to adjust the preexisting maternal risk of PE with sufficient specificity and sensitivity for clinical use. As with screening for aneuploidies, the best screening strategies for PE involve several parameters in combination. 9 Next, we describe the main factors used in these algorithms, alone and in combination.

## Maternal characteristics

The use of information from maternal pathological history and gestational history in the assessment of risk for PE offers a reasonable performance and is still proposed in some national guidelines. The Institute for Health Care and Clinical Excellence (NICE) PE screening guidelines were investigated in a prospective study, 10 describing the possibility of a detection rate of 90% for preterm PE and 89% for term PE, at the expense of a 64.1% false-positive rate. The authors demonstrate that these same factors combined in an algorithm derived from multivariate analysis produce a detection rate of 37% for early-onset PE and 28.9% for late-onset PE, and a 5% false-positive rate. The limitations of using maternal factors alone to predict PE in primigravidae were well illustrated in the prospective multicenter SCOPE study in which an algorithm was developed; it detected 37% rate of PE for a 10% false-positive rate and 61% for a 25% false-positive rate. 11

## Biomarkers

A wide range of potential biomarkers for PE has been identified in the maternal circulation, reflecting the complex pathogenesis of this condition. 12 However, no biomarker has demonstrated sufficient predictive value to be of clinical utility if used alone. 13 Instead, they appear to be more valuable in combination with other parameters.

## Mean blood pressure

Mean arterial pressure (MAP) is calculated by dividing the sum of systolic blood pressure with twice the diastolic blood pressure divided by three. A prospective study of 5,590 women with singleton pregnancies identified that a combination of maternal risk factors and MAP measured at 11-14 weeks' gestation was more predictive of PE than its use alone. 14 In this study, the combination of maternal history and PAM identified 62.5% of PE cases at a 10% false positive rate. The combination of these two factors is currently the basis of virtually all PE screening strategies.

## Doppler velocimetry of the uterine arteries

The abnormal placentation that characterizes PE is associated with increased resistance in the uteroplacental circulation. Based on this premise, the analysis of uterine artery Doppler velocimetry in the risk assessment for PE has been extensively studied, initially in the second trimester and later in early pregnancy. Doppler velocimetry evidence of this resistance includes a qualitative and quantitative assessment of flow. In the qualitative assessment, a protodiastolic notch is observed in the waveform. Quantitative assessment demonstrates the increase in the pulsatility index (PI) of this vessel. 15 Current risk calculation algorithms preferentially use quantitative assessment because the PI value is a continuous variable objectively measured. 16

The ability to predict PE using uterine artery Doppler velocimetry is quite limited, and the performance of this parameter is better in the second trimester and in the identification of early-onset PE. First-trimester uterine artery Doppler sensitivity in predicting PE was 26% (95% confidence interval [CI]: 24-29) and specificity was 91% (95% CI: 91-91) in a meta-analysis involving 11 studies. 17 Studies have suggested that uterine artery Doppler may be more predictive if performed sequentially in the first and second trimester. 18 However, such an approach would prevent the timely early initiation of prophylaxis.

## Biochemical markers

Several biochemical markers have been described in the prediction of PE, but only two (placental growth factor [PlGF] and pregnancy-associated plasma protein A [PAPP-A]) have shown some discriminatory power and have been used. The PlGF is a glycosylated dimeric glycoprotein secreted by trophoblast cells and part of the angiogenic vascular endothelial growth factor (VEGF) family. This isolated biomarker has a detection rate of 55% and 33% for the identification of early- and late-onset PE, respectively for a false-positive rate of 10%. 19 The PAPP-A is an insulin-like growth factor binding protein of the metalloproteinase secreted by the syncytiotrophoblast that plays an important role in placental growth and development. A maternal concentration of PAPP-A below the 5 ^th^ percentile is associated with the risk of developing PE, with a detection rate of 16% and a false-positive rate of 8%. 20

## Multiparametric tests

A systematic review evaluating PE screening models indicated that among 16 models validated in four studies, only five (four first trimester models and one second trimester model) were considered to have statistically acceptable discriminatory characteristics. 21 The use of a multivariate logistic regression algorithm, a combination of maternal factors, MAP, uterine artery PI, maternal serum PAPP-A and PlGF at 11-13 weeks' gestation allowed the detection of rates of 93% and 36% for the prediction of early- and late-onset PE, respectively, for 5% false positives. 22 23 The largest study to date on the development of the first-trimester combined test using the concurrent risk model was reported by Tan *et al* . 24 In this study, from a 1 in 100 risk cutoff for PE in white women, the positive screening rate was 10% and the detection rates of preterm and full-term PE were 69% and 40%, respectively.

## Validation of models in the Brazilian population

The Fetal Medicine Foundation (FMF) prediction models were prospectively evaluated in several countries, with similar results, including Brazil, 25 and were recently approved by the International Federation of Gynecology and Obstetrics (FIGO) in the screening of PE. 26 A study conducted in Brazil using the FMF model based on maternal characteristics and PAM showed a detection rate of 67% of preterm PE cases, at a false positive rate of 10%, a positive predictive value of 17% and negative predictive value of 99%. 3 The performance of universal screening is important, always using a risk calculation model, but the parameters adopted will depend on the availability of each service.

## Prevention of preeclampsia

### What interventions reduce the risk of preeclampsia?

- Acetylsalicylic acid (ASA): Aspirin for Evidence-Based Preeclampsia Prevention (ASPRE) was a randomized, double-blind, placebo-controlled trial that identified patients at high risk for PE at 11-14 weeks' gestation using the combined screening test of the FMF; then, ASA (150 mg daily at bedtime) was compared with placebo in those defined as high risk from 11-14 weeks' to 36 weeks' gestation. This landmark study showed a significant 62% reduction for preterm PE. There was no reduction in the incidence of PE at term, but this may be due to a delay in the onset of the disease, resulting in a shift in the distribution to the right. 27
- Physical exercise: Moderate intensity exercise (enough to increase the heart rate and allow you to speak but not sing) performed for at least 140 minutes per week can reduce the risk of PE. A systematic review of 3,322 women showed that exercise reduced the risk of PE in 41% of them, without adverse fetal effects. 28
- Induction of labor: A study investigating 6,106 low-risk nulliparous women showed that induction of labor at 39-39 weeks and 4 days of pregnancy reduced the risks of gestational hypertension and PE compared to expectant management. 29
- Low molecular weight heparin (LMWH): There is no recommendation for the use of LMWH to prevent PE. 30 31 Only the *Society of Obstetricians and Gynecologists of Canada* (SOGC) discusses heparin as an option in women with a history of placental complications. 32 The indication of LMWH should be restricted to women with other comorbidities who require anticoagulation during pregnancy, such as antiphospholipid syndrome. A possible beneficial effect of the combination of low doses of ASA and LMWH in preventing PE in this high-risk group is unclear. 33
- Calcium supplementation: The evidence for general calcium supplementation for all women in preventing hypertensive disorders is conflicting. In a 2014 meta-analysis, daily calcium supplementation of ≥ 1 g in the second half of pregnancy showed a significant 55% reduction for PE, particularly for women on a low-intake diet (13 trials, 15,730 women: relative risk (RR): 0.45; 95% CI: 0.31-0.65; I2 = 70%). 34

### When is ASA indicated for the prevention of preeclampsia?

Using the FMF combined screening algorithm, the ASPRE study proposed a risk cutoff of 1:100 to define the high-risk group, which led to a detection rate of 77% for a positive screening rate of 11%. 27

### Is the use of ASA safe in pregnancy?

The use of ASA during pregnancy appears safe for both the mother and the fetus. Treatment with ASA did not show an increased risk of congenital malformations and had no negative effect on fetal development or bleeding complications in the neonatal period. 35 36 37 Despite side effects such as minor vaginal bleeding and gastrointestinal symptoms, which occur in approximately 10% of users , there is no evidence of an increased risk of major maternal bleeding or association with placental abruption. 27 Concerns about premature closure of the fetal ductus arteriosus have never been confirmed. However, there is a lack of data on possible side effects and long-term outcomes when ASA is prescribed on a large scale to low-risk patients. 27

### When to start ASA for patients at high risk for preeclampsia?

Most trials using ASA to prevent placental complications started treatment at or after 12 weeks' gestation. There is current convincing evidence that the strongest reduction in premature PE is achieved with initiation of therapy before 16 weeks' gestation. 38 However, the incidence of PE can still be positively influenced when ASA is started only after 16 weeks' gestation and given its safety profile, high-risk women who present for antenatal care after 16 weeks may still benefit from prophylaxis. Note that this aspect has been controversially discussed in the literature, and the maximum prophylactic effect seems to occur when ASA is started early. 39

### What is the optimal dose of ASA to prevent preeclampsia?

The most commonly evaluated daily doses of ASA range from 60 to 162 mg. However, in vitro and in vivo studies have shown that the optimal dose is ≥ 100 mg per day. 38 40 It also appears that there is a clear dose-dependent effect. In a study published by Caron *et al.* , 41 at a daily dose of 81 mg, 121 mg, and 162 mg, 30%, 10%, and 5% of subjects were classified as non-responders, respectively. Therefore, doses below 100 mg should be avoided, 27 although direct comparisons of different dose regimens in randomized trials are not available. In Brazil, ASA at a dose of 100 mg is widely available and inexpensive, hence an interesting option is the use of one and a half ASA pill to prevent PE in our country. It is important to emphasize the need to discard the residual portion of the tablet, as its use in the following day is not supported in the literature.

### When should patients stop taking ASA?

In most RCTs and meta-analyses, a significant increase in major bleeding complications has not been found and in the absence of other anticoagulants, neuraxial blockade is not contraindicated. 27 42 The ASPRE study discontinued ASA use at 36 weeks' gestation, but treatment until delivery is considered safe. There are no studies evaluating if stopping prophylaxis at an earlier gestational age would have similar efficacy.

### What to do with patients at high risk for preeclampsia who report a known allergy to ASA?

In patients with a known urticarial allergic reaction to ASA or other contraindications such as bleeding disorders or severe asthma, ASA should not be used. Patients at high risk for PE who cannot take ASA may benefit from calcium supplementation or LMWH in specific cases. These interventions should be considered on a case-by-case basis after appropriate counseling and risk-benefit assessment.

## Final considerations

Preeclampsia is a condition that results in high maternal and perinatal morbidity and mortality worldwide, with a more severe impact on developing countries such as Brazil. Considering the availability of efficient tools for early screening and low-cost prophylaxis, we recommend: (1) universal screening of PE in the first trimester using a risk calculation model; (2) use of ASA at a dose ≥ 100 mg for PE prophylaxis in patients with high-risk screening.

National Commission Specialized in ultrasound in GO of the Brazilian Federation of Gynecology and Obstetrics Associations (Febrasgo)

President:

Eduardo Becker Júnior

Vice-president:

Heron Werner Júnior

Secretary:

Sergio Kobayashi

Members:

Adriana Gualda Garrido

Anselmo Verlangieri Carmo

Fabricio da Silva Costa

Fernando Maia Peixoto Filho

Guilherme de Castro Rezende

Joffre Amim Junior

Jorge Roberto Di Tommaso Leao

Luciano Marcondes Machado Nardozza

Luiz Eduardo Machado

Manoel Alfredo Curvelo Sarno

Patricia El Beitune

Pedro Pires Ferreira Neto
